# Quasicrystalline phase-change memory

**DOI:** 10.1038/s41598-020-70662-2

**Published:** 2020-08-13

**Authors:** Eun-Sung Lee, Joung E. Yoo, Du S. Yoon, Sung D. Kim, Yongjoo Kim, Soobin Hwang, Dasol Kim, Hyeong-Chai Jeong, Won T. Kim, Hye J. Chang, Hoyoung Suh, Dae-Hong Ko, Choonghee Cho, Yongjoon Choi, Do H. Kim, Mann-Ho Cho

**Affiliations:** 1 Material Research Center, SAIT, Samsung Electronics, Suwon, 16678 Republic of Korea; 2 Department of Materials Science and Engineering, Yonsei University, Seoul, 03722 Republic of Korea; 3 Department of Physics, Yonsei University, Seoul, 03725 Republic of Korea; 4 Department of Physics and Astronomy, Sejong University, Seoul, 05006 Republic of Korea; 5 Department of Optical Engineering, Cheongju University, Cheongju, 28503 Republic of Korea; 6 Advanced Analysis Center, Korea Institute of Science and Technology, Seoul, 02792 Republic of Korea

**Keywords:** Energy, Inorganic chemistry, Materials chemistry, Electronic devices, Computational nanotechnology, Structure of solids and liquids

## Abstract

Phase-change memory utilizing amorphous-to-crystalline phase-change processes for reset-to-set operation as a nonvolatile memory has been recently commercialized as a storage class memory. Unfortunately, designing new phase-change materials (PCMs) with low phase-change energy and sufficient thermal stability is difficult because phase-change energy and thermal stability decrease simultaneously as the amorphous phase destabilizes. This issue arising from the trade-off relationship between stability and energy consumption can be solved by reducing the entropic loss of phase-change energy as apparent in crystalline-to-crystalline phase-change process of a GeTe/Sb_2_Te_3_ superlattice structure. A paradigm shift in atomic crystallography has been recently produced using a quasi-crystal, which is a new type of atomic ordering symmetry without any linear translational symmetry. This paper introduces a novel class of PCMs based on a quasicrystalline-to-approximant crystalline phase-change process, whose phase-change energy and thermal stability are simultaneously enhanced compared to those of the GeTe/Sb_2_Te_3_ superlattice structure. This report includes a new concept that reduces entropic loss using a quasicrystalline state and takes the first step in the development of new PCMs with significantly low phase-change energy and considerably high thermal stability.

## Introduction

**Figure 1:**
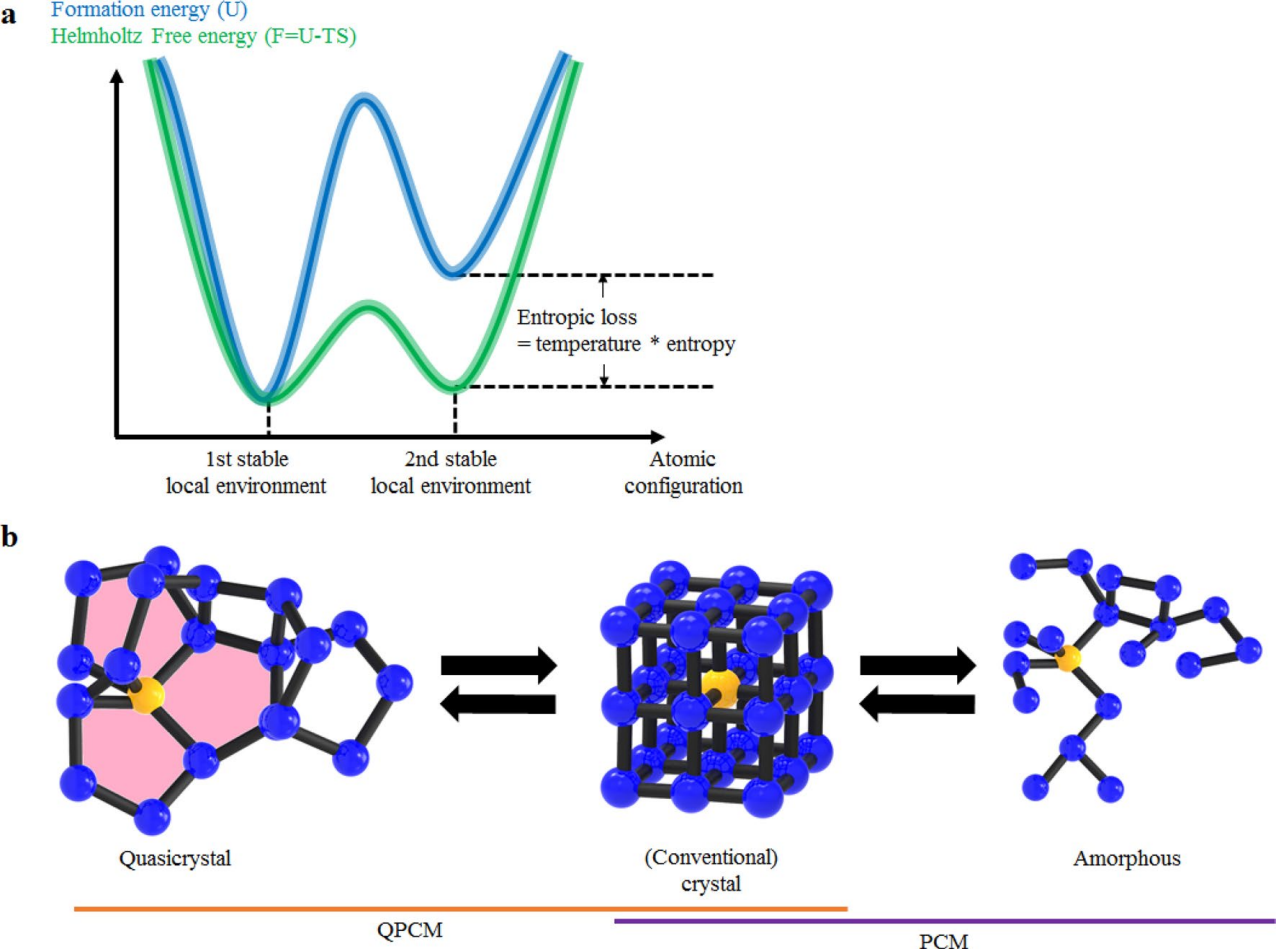
Scheme of phase-change process of quasicrystal phase-change material (QPCM) (**a**) Energy hierarchy of local environments of PCM (**b**) phase change process of QPCM and PCM.

Recently, phase-change materials (PCMs) have been utilized as a 3D Xpoint technology, which is a type of nonvolatile memory based on the reversible amorphous-to-crystalline phase-change process of chalcogenide materials. Memory switching devices were first proposed by Ovshinsky in 1968^1^, and after 20 years, GeTe–Sb_2_Te_3_ pseudobinary compounds (GST) were discovered by Yamada et al^2^*.* It is interesting to note that the phase-change properties of relevant materials, such as phase-change energy, speed, repeatability, and thermal stability were sufficient to commercialize optical storage devices^2–5^. From a microscopic perspective, as phase-change processes can be driven by local environment evolution between first and second stable local environments, phase-change properties are determined by the energetic stability of the second stable local environment relative to that of the first stable local environment, as depicted in Fig. 1a^6–13^. Unfortunately, the development of new PCMs with low phase-change energy and high thermal stability has not yet met the requirements for widespread commercialization in the automobile industry^14^ because the problem of trade-off between thermal stability and phase-change energy has not been solved yet in the amorphous-to-crystalline phase-change processes. In other words, destabilization of the second stable local environment leads to a simultaneous decrease in thermal stability and phase-change energy. The trade-off between phase-change energy and thermal stability has now been resolved by reducing entropic loss of phase-change energy, utilizing a crystalline-to-crystalline phase-change process, as depicted in the GeTe/Sb_2_Te_3_ superlattice structure^15^. The phase change in the superlattice structure can be achieved via one-dimensional atomic movements instead of 3-dimensional ones in a GST alloy, which results in a decrease in phase-change energy of up to one order of magnitude while retaining the extent of thermal stability, compared to GST alloys^6,15^.

In contrast, a paradigm shift in atomic crystallography has been recently affected by quasi-crystals (QCs) that comprise a new type of atomic ordering without linear translational symmetry, as depicted in Fig. 1b. These structures have discrete point-group symmetry that is not allowed for periodic systems of conventional crystals^16–18^. This unique crystal structure leads to successful accomplishments with regard to specific electrical properties of QCs such as polarizer, superconductor, and thermoelectric materials^19–21^. However, although entropic loss during phase-change processes can be excluded when utilizing the phase-change process between QC and approximant crystal (AC), phase-change properties for memory devices utilizing QC have not yet been satisfactorily investigated, unlike those for the phase changes between amorphous and crystal states, which have been established as promising for the development of new PCMs. In this study, we investigated the phase-change properties of Al–Mn–Si, which has been reported to be in the QC and AC phase, such as the dependence of resistivity on annealing temperature during the phase-change process, differential scanning calorimetry (DSC), structural properties [X-ray diffraction (XRD)], and transmission electron microscope (TEM). Finally, electrical memory switching characteristics were confirmed in memory cells using electrical pulse switching. Further, we introduce a novel class of PCMs based on a quasicrystalline-to-approximant crystalline phase-change process, whose phase-change energy and thermal stability are enhanced to a higher extent than those of the GeTe/Sb_2_Te_3_ superlattice structure. This is accomplished by suppressing entropic loss, where fast phase-change speed (< 10 ns) and symmetricity and linearity for neuromorphic computing are also secured, simultaneously. Our report reduces entropic loss by utilizing QCs and opens a door to the development of new PCMs with super-low phase-change energy and super-high thermal stability.

## Results

**Figure 2:**
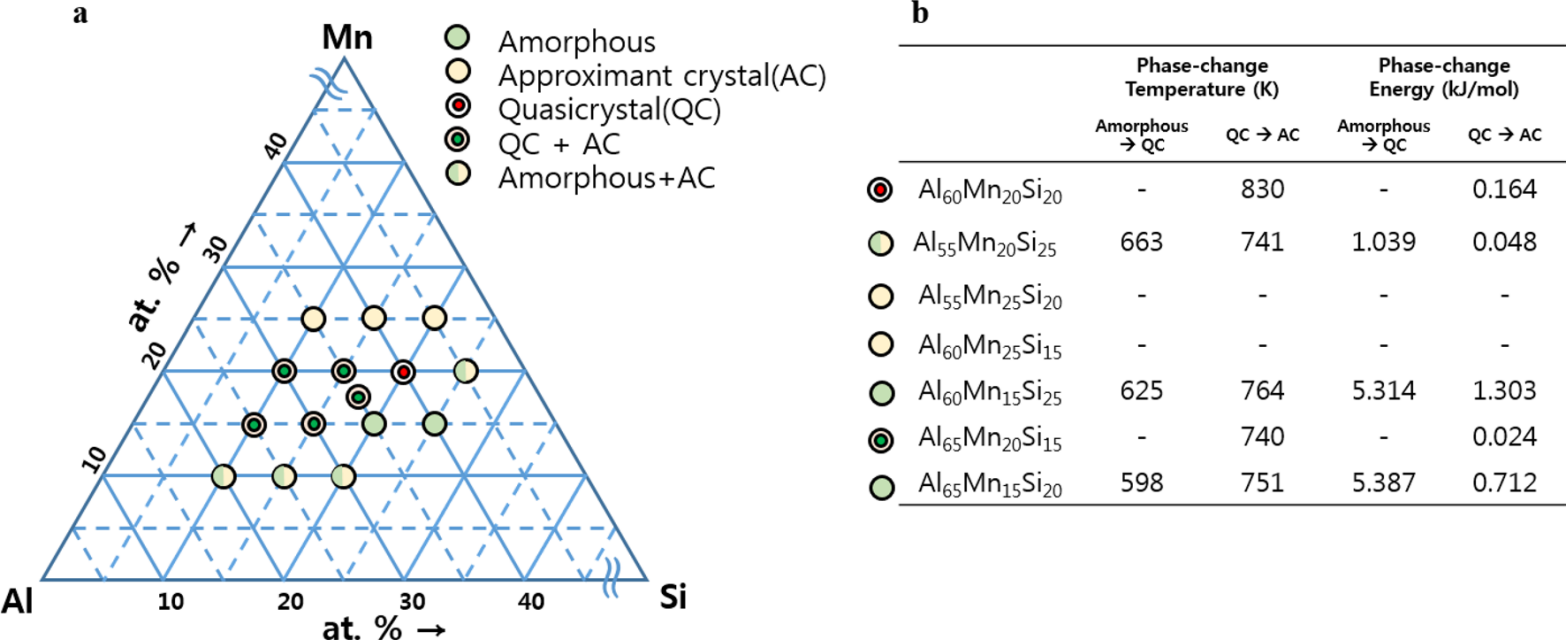
Phase selection in the Al–Mn–Si alloy system. (**a**) Phase diagram of melt-quenched ribbon of Al–Mn–Si ternary alloys. Among the alloys investigated, only Al_60_Mn_20_Si_20_ consisted almost solely of the QC phase. (**b**) Phase-change temperature and energy of Al–Mn–Si ternary alloys.

There are some prerequisites to utilize a QC as a PCM for electronic memory; e.g., the alloy must have both stable and metastable states, and there must be a difference between the electrical resistances of the two phases^22^. Because approximant crystal (AC) has linear translational symmetry and differential electrical resistance, in contrast to QC, both phases, AC and QC, can be utilized for the PCM. The Al–Mn–Si alloys meet the prerequisites for PCM among the various alloy systems capable of forming the QC and AC phases^23–25^. Fig. 2a illustrates a phase diagram of a ternary compound of melt-quenched (10^6^ K/s) Al–Mn–Si alloys classified into three phases—amorphous, QC, and AC—obtained via XRD, as depicted in Supplementary Fig. S3. It should be noted that activation energies which correspond to the phase-changes from amorphous to QC and from QC to AC state of Al–Mn–Si alloys such as Al_55_Mn_20_Si_25_, Al_60_Mn_15_Si_25_, Al_65_Mn_15_Si_20_ are comparable with that from amorphous to crystalline state of Ge–Sb–Te (2.34 eV) which supports that amorphous and QC phase is stable enough to sustain each phase at room temperature even though they are not the most stable state^26^. The AC phase is dominant when the proportion of Mn in the Al–Mn–Si alloy exceeds 25%, and the QC phase dominates when it lies in the range of 15–20%, where Al_60_Mn_20_Si_20_ (AMS) is the only QC phase without amorphous or AC states. Figure 2b depicts phase-change temperatures and energies of melt-quenched Al–Mn–Si alloys of various compositions, which reflects the trade-off between thermal stability and phase-change energy. The Al–Mn–Si ribbon, whose composition includes an amorphous phase, exhibits high phase-change energy compared to the others, indicating the high involvement of energy of entropic loss during the phase-change process. Via the screening of materials in Al–Mn–Si alloys, the melt-quenched AMS, which has a single QC phase, is studied because the phase-change energy is reduced by suppressing the energy of entropic loss. Moreover, single phase can be very usefully applied to an electrical PCM, because co-existence of both phases with different electrical characteristics degrades phase-change properties and the electrical resistance of cells.

**Figure 3:**
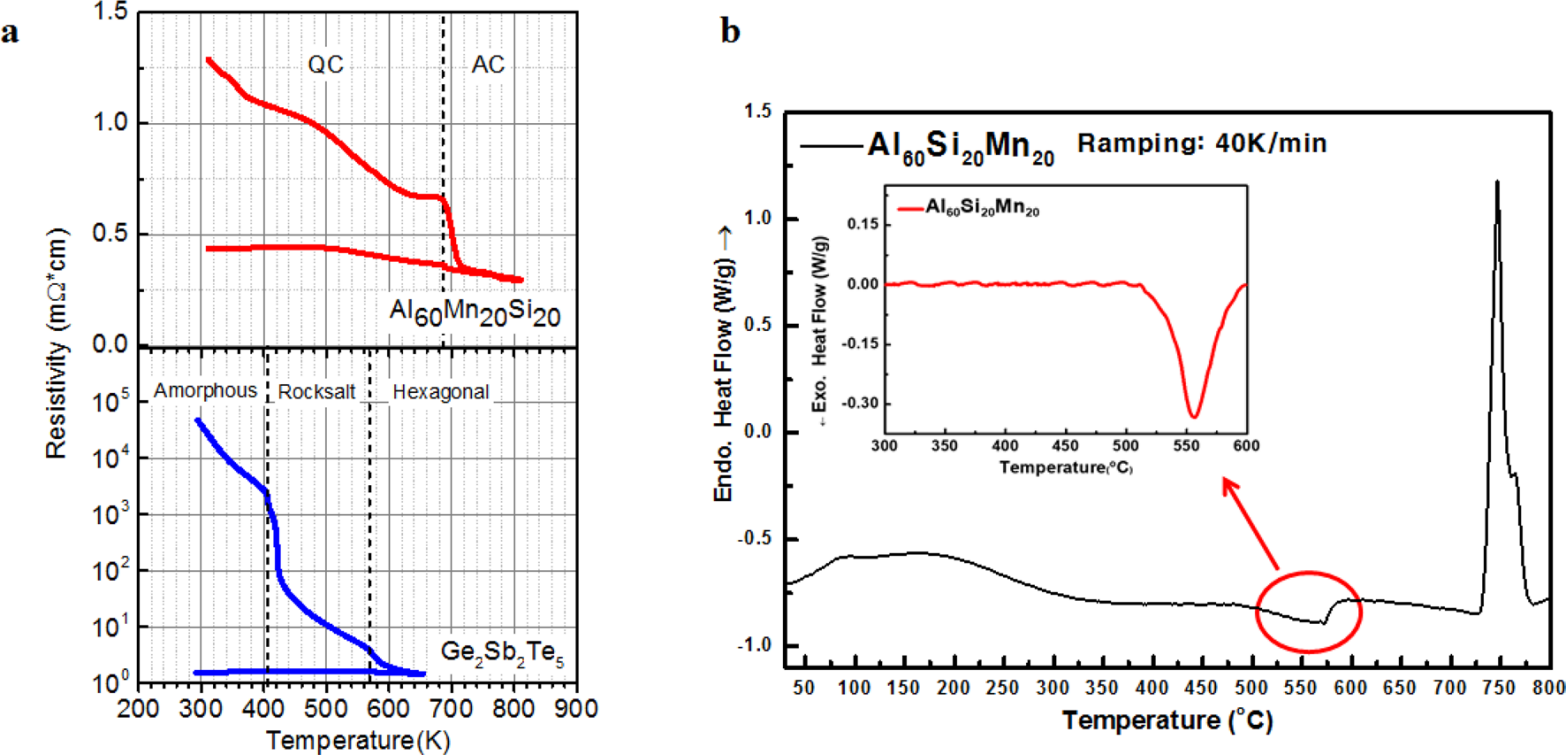
Resistance–temperature curve and DSC analysis. (**a**) Resistivity versus temperature for Al_60_Mn_20_Si_20_ and Ge_2_Sb_2_Te_5_ (GST) thin-films heated at 30 K/min. As-deposited amorphous GST thin film and Al_60_Mn_20_Si_20_ QC thin films which were annealed up to 623 K for 2 h with thicknesses 50 and 100 nm are shown in upper and lower panel, respectively. 50 nm and 100 nm films are colored red and green respectively. (**b**) DSC analysis for the thermal behavior of the QC phase. Low temperature DSC trace (up to ~ 900 K) obtained from the as-melt-spun Al_60_Mn_20_Si_20_ alloy during continuous heating at 40 K/min. The DSC trace shows an exothermic reaction corresponding to the QC to AC phase-change process (onset temperature: ~ 793 K). Inset: high temperature DSC trace (up to ~ 1,100 K) showing an endothermic reaction corresponding to the AC to liquid melt phase-change process (onset temperature: ~ 1,003 K).

Figure 3a records the dependence of electrical resistivity on the annealing temperature of AMS, where the QC-to-AC phase-change process is included. As the temperature is increased, the resistivity of the AMS gradually decreases; i.e. the coefficient of dependence is negative for QC^27^, indicating that the bonding type of AMS is covalent, rather than metallic, despite the high concentration of Al (60%). Resistivity of the 50 nm-thick AMS film on SiO_2_ drops sharply at approximately 700 K, which can be attributed to the phase-change process from the QC to the AC phase. This temperature is 150 K higher than the crystallization temperature of Ge_2_Sb_2_Te_5_ (GST), indicating that the relatively high crystallization temperature of AMS meets the requirements of commercialization in the automobile industry. In other words, the relatively high crystallization temperature of AMS is advantageous in the devices that request high reliability at temperatures above 500 K. Figure 3b presents the DSC results for a AMS ribbon of 20 µm thickness, whose phase-change process from QC to AC occurs at 830 K and that of AC to liquid occurs at 1,010 K. The increase in phase-change temperature from QC to AC for the relatively thick AMS film is consistent with our result: i.e., the temperatures of phase-change from QC to AC for 50 nm, 100 nm, and 200 nm films are 685 K, 705 K, and 760 K, respectively. This can be attributed to the relatively large-scale ordering during phase-change process from QC to AC compared to that in conventional PCMs. Despite this elevated phase-change temperature, phase-change energy from QC to AC of AMS is observed to be significantly low—0.164 kJ/mol—which is twenty times lower than that of GST (3.9 kJ/mol). The remarkably low phase-change energy of AMS caused by the suppression of entropic loss during QC-to-AC phase-change process indicates a productive direction for designing new PCMs. In other words, the serious trade-off problem that the decrease in the phase-change energy simultaneously reduces thermal stability caused by the destabilization of amorphous phases in conventional PCM devices can be solved by implementing QC-to-AC phase-change processes. Reliability was quantified via increases in crystallization temperature (up to 150 K), as well as by decreases in phase-change energy (by up to twenty times) caused by entropic loss. The phase change characteristics of AMS are comparable with that of the interfacial phase-change memory (iPCM), where the [GeTe/Sb_2_Te_3_]_n_ superlattice reduces phase-change energy to a greater extent than GeTe–Sb_2_Te_3_ pseudobinary alloys because three dimensional atomic movement is restricted to one dimensional atomic movement.

**Figure 4:**
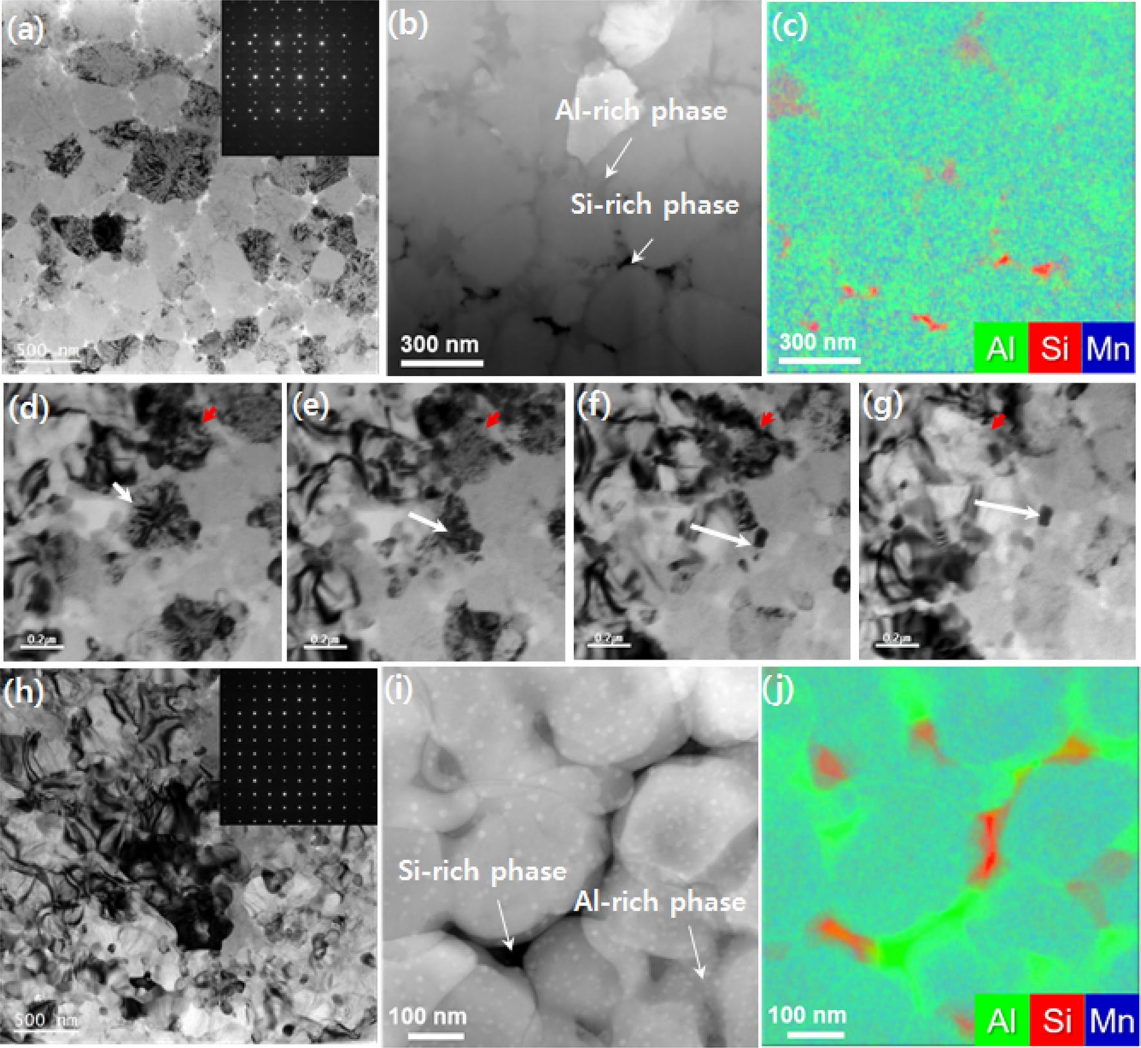
In-situ TEM analysis for the QC to AC phase-change process. (**a**)–(**c**) TEM images obtained from as-melt-spun Al_60_Mn_20_Si_20_ alloy: (**a**) BF TEM image (inset: selected area diffraction pattern, SAED pattern showing twofold symmetry zone of QC phase); (**b**) high angle annular dark field–scanning transmission electron microscope (HAADF-STEM) image; and (**c**) corresponding elemental map. (**d**)–(**g**) BF TEM images showing QC-AC phase-change process at 723 K in TEM: (**d**) QC phase before heating (marked with white and red arrows); (**e**) AC phase starts to grow after nucleation at the grain boundary region of the QC phase (marked by white arrow) and transforms abruptly from QC to AC (marked by red arrow); and (**f**) and (**g**) AC phase continues to grow replacing the QC phase and the phase-change process is finally complete. (**h**)–(**j**) TEM results after the QC to AC transition at 723 K: (**h**) BF TEM image and SAED pattern showing [001] zone of the AC phase; (**i**) HAADF-STEM image; and (**j**) the corresponding elemental map.

Figure 4 presents the direct observation of the phase-change process of AMS from QC to AC with in-situ TEM on annealing from room temperature to 723 K. Figure 4a depicts a bright field (BF) TEM image of QC AMS at room temperature, where the average grain size of the QC phase was observed to be ~ 300 nm. twofold symmetry of selected area electron diffraction (SAED) patterns in melt-quenched AMS, as depicted in the inset of Fig. 4a serves as the strongest evidence for the non-standard symmetry in QC, which cannot be explained using linear translational symmetry. As mentioned in the phase diagram in Fig. 1, melt-quenched AMS exists as QC and no amorphous rings can be observed in SAED patterns. In further detail, although Al- and Si-rich phases were present at the grain boundary of the QC phase, the effect was marginal and the region did not expand with repeated phase-change processes. The composition of the QC phase was measured using energy dispersive x-ray spectroscopy (EDS) and was found to be 58 at.% Al, 19 at.% Si, and 23 at.% Mn. Therefore, enrichment of Al and Si occurs at the growth front of the primary QC phase, which leads to the formation of Al-rich (62 at.% Al, 19 at.% Si, and 20 at.% Mn) and Si-rich (37 at.% Al, 46 at.% Si, and 16 at.% Mn) secondary phases in the grain boundary region after solidification. The QC-to-AC phase-change process in melt-quenched Al_60_Si_20_Mn_20_ alloy was traced by real time imaging using in-situ TEM, as depicted in Supplementary Video SV1. Figure 4d-g presents a series of BF TEM images, including the QC-to-AC phase-change process, carried out at 723 K, as indicated by the red and white arrows. The grain indicated by a red arrow transforms abruptly, as depicted in Fig. 4f,g, while the QC/AC phase boundary, marked by a white arrow, moves continuously across the grain as the transformation proceeds, as depicted in Fig. 4d-g. Figure 4h–j present the TEM images after the *in-situ* annealing process, where the SAED pattern exhibits fourfold symmetry, indicating AC with linear translational symmetry. No severe phase separations were observed, as is apparent from the EDS data presented in Fig. 4j. The average grain size during the AC phase after the phase-change process was ~ 300 nm, which is approximately the same as that during the QC phase.

**Figure 5:**
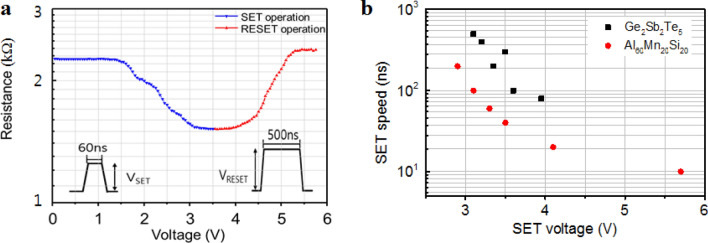
Resistance–temperature curve and cell operation performance. (**a**) Resistance–voltage curves for the set and reset operation processes in the Al–Mn–Si alloy system. (**b**) Set operation speed of Al–Mn–Si and GST.

Figure 5a,b record the electrical device operation characteristics of AMS in a T-shaped test cell, where the diameter of the bottom electrode contact (BEC) is 300 nm, which is much larger than that of the commercialized ones. Figure 5a illustrates the resistance–voltage (R–V) curves for the PCM cells utilizing the QC and AC phases of Al_60_Mn_20_Si_20_ processes by applying voltage pulses between 60 and 500 ns. The resistance changes after the application of the electrical ns pulse, where the phases of the high resistance state (HRS ~ 2.3 kΩ) and the low resistance state (LRS ~ 1.5 kΩ) correspond to QC and AC, respectively. This clearly demonstrates electrical operating memory property based on electrical resistance contrast. The operation characteristics indicate that the electrical device properties of linearity and symmetry for neuromorphic computing are improved, compared to those of GST and transition metal doped Sb_2_Te_3_. To confirm the set speed of the Al–Mn–Si alloy, the operation was investigated repeatedly under electrical pulses of various durations. The operation speed and voltage of electrical pulses for the phase-change process from QC to AC were measured at various applied voltages, as depicted in Fig. 5b. The set operation speed for the QC-to-AC phase-change process in Al–Mn–Si is higher than that during the amorphous-to-crystalline conversion in GST over the entire applied voltage range, e.g., the set speed of the PCM cell with Al–Mn–Si alloy was 10 ns, whereas that with GST, which has been used in this study, was 80 ns; thus, better phase-change properties are expected in the latter case under optimized bottom electrode contact and pulse generator. The improved set operation speed can be attributed to the suppression of entropic loss because of the reduced atomic movements required for the phase-change process in AMS^26^.

## Discussion

This study introduces a unique phase-change process from QC to AC, without considering amorphous-to-crystalline phase-change represented by GST. Using Al–Mn–Si systems as a prototype for developing the promising candidates for the next-generation is a novel concept in the context of PCMs. The amorphous-to-crystalline phase-change processes occur in chalcogenide semiconductor materials via nucleation and crystal growth. The nucleation growth process is determined by competing capillary forces and driving forces, which resist the formation of new interfaces and drive the growth of a new and more stable phase, respectively. Stochastic nucleation rate is highly affected by the interfacial Gibbs free energy ($\upgamma$) and the difference between the bulk free energies of the new and parent phases. Following classical nucleation theory^28^, the nucleation rate increases very rapidly as the interface energy decreases because the energy barrier for nucleation is proportional to γ^3^. The nucleation process is an important step in determining phase-change speed because the formation of critical nuclei requires an incubation delay of a period between tens of microseconds and sub-microseconds^26^. This delay depends on the melt-quenched state of Ag-In–Sb–Te or Ge_2_Sb_2_Te_5_ because remnant nucleation sites can be controlled by the melt-quenching process^29,30^. Therefore, the stability of a reset state under a memory operation is critically affected by the remnant nucleation sites during the amorphous phase.

In contrast, when metastable QC is formed by rapid solidification, it can be characterized by an uneven distribution of quenched phasons, which is a quasiparticle representing the movements of atoms just like phonon, and chemical disorder^17^ The amount of disorder in metastable QC is known to increase as the cooling rate increases and it is also dependent on the alloy composition. Finally, metastable QC can be transformed into a stable AC at high temperatures, as recorded in the reported result that Al–Mn metastable QC with the same basic structural units, called the Mackey cluster, was transformed with little changes in composition^31^. Therefore, the interface energy between the newly formed AC and QC matrices is expected to be small, even though the exact value of the interface energy is not yet known. The interface energy between the QC and AC phases is expected to be much smaller than that between the crystalline and amorphous phases of chalcogenide phase-change materials. In particular, the small heat for transformation obtained in this study, as depicted in Fig. 3b, indicates that the bonding nature of the QC phase is similar to that of the AC phase, supporting low interface energy. It has been reported that the heat emitted during amorphous-to-crystalline phase-change process in AgIn-Sb_2_Te_1_ and Ge_2_Sb_2_Te_5_ is 4.3 or 3.9 kJ/mol^32^. This is two orders of magnitude greater than that corresponding to the QC-to-AC phase-change process in melt-spun Al_60_Mn_20_Si_20_ alloy (0.164 kJ/mol), as depicted in Fig. 4. This indicates that the QC phase has an internal structure similar to that of the AC phase, rather than that of the amorphous phase. Therefore, a high nucleation rate is expected for the QC-to-AC phase-change process. The detailed microscopic mechanism for this transformation has not yet been clarified and further study is required.

As mentioned, the metastable QC contains randomly distributed phason defects. Some of the local domains containing linear phasons in the as-quenched metastable QC phase may have the same structure as the AC phase. Thus, any continuous transformation, obtained via the extension of the existing domain, contains a few AC unit cells randomly distributed in the domain, as depicted in Fig. 4d–g. The local domain can then act as a nucleus because the critical size of nuclei is small. Therefore, the low interface energy between domains consisting of the same type of building blocks (clusters) and the negligible incubation time required to form critical nuclei during the QC-to-AC phase-change process leads to fast phase-change speed and small phase-change energy. Further, the extension of the periodic structure only requires limited rearrangement of the atoms. Finally, the high phase-change speed achieved by the method in this study can be attributed to the high nucleation rate that arises as a result of the low interface energy between QC and AC and the activated site of pre-existing quenched-in nuclei in the local domain. Considering that the heat from the transformation is sensitive to the alloy composition, there may be further scope to enhance the transformation kinetics by optimizing the alloy composition. Therefore, the results of this study indicate that the Al–Mn–Si QC phase could make significant contributions to the next generation of PCMs, for which fast switching speeds and long cyclic lifetimes are imperative. In order to evaluate phase-change properties, such as crystallization temperature, resistance on–off ratio, phase-change speed, and energy of QC-to-AC phase-change process for the Al_60_Mn_20_Si_20_ alloy, R-T curves, DSC, and electrical cell operation performances were recorded and have been presented in Figs. 3 and 5. From the comparison of the R-T curve for the Al–Mn–Si alloy with that for the GST, as depicted in Fig. 3a, the phase-change temperature from QC to AC can be observed to be 700–726 K, depending on the film thickness, which is much higher than ~ 425 K in the case of GST. The higher phase-change temperature indicates that the QC has better thermal stability and therefore better retention can be expected on the corresponding PCM cell. The higher temperature can be attributed to the relatively low atomic number of composite elements in AMS compared to those of conventional PCMs, such as GST and Ag–In–Sb–Te. This is consistent with reports that the presence of doping light elements (Ti or Sc) in Sb_2_Te_3_ increases phase-change temperature due to the formation of strong bonding with the light element. The faster phase-change speed of AMS compared to GST can be explained by the low interfacial energy and negligible incubation times during nucleation; i.e., the quenched-in nuclei process accompanying minimal structural and compositional changes during the QC-to-AC phase-change process could be accomplished via the low configurational entropy loss.

Additionally, origins of the contrast between the electrical resistances of QC and AC for AMS are studied via optical conductivity obtained by THz-TDS, as depicted in Supplementary Fig. S3, for developing new PCMs that utilize QC-t-AC phase-change processes. The DC limit of electrical resistance of THz-TDS is consistent with our results of the dependence of resistance on temperature during the annealing and cooling processes. The optical conductivity of QC and AC AMS can be fitted with Drude model. This is because the carrier concentration is higher and the mobility is lower in AC, compared with those in QC, and the electron plasma frequency (*ω*_*p*_) and electron scattering rate (γ_0_) are *ω*_*p*_ = 0.5 and 0.7 eV for QC and AC, respectively; γ_0_ = 9 × 10^12^ and 6 × 10^12^ s^−1^ for QC and AC, respectively. The difference in the free carrier density is consistent with reports that the metallic-covalent bonding conversion is a characteristic feature of Al-based iQC systems^33^. When QC systems include transition metals (TMs), such as Mn, strong covalent bonds are more likely to be observed at TM-X sites (X = Al or Si in our system)^34,35^. Such covalent bonding is reported to be weaker in the AC phase than in the QC phase^34,35^. Further, the scattering rate of ACs is lower than that of QCs due to the percolation path of free carriers and the longer mean free path during which phason strain is relaxed^36^. This agrees with the increase in mobility observed in the periodically ordered AC structure. The higher free carrier density and carrier mobility may result in changes in resistivity that are three times higher in the QC phase than in the AC phase.

## Conclusions

Phase-change properties of AMS, which undergoes solely QC phase after the melt-quenching process, were studied to investigate their applicability in PCMs. The phase change characteristics of Al–Mn–Si alloys were investigated using electrical cells of PCMs based on phase-change temperature and energy of melt-quenched ribbons at speeds of 10^6^ K/s. Super-low energy consumption for phase-change, super-high thermal stability, fast phase-change speed, and low resistance drift of AMS could be accomplished, which can be attributed to the suppression of energy of entropic loss compared to that during GST and conventional PCMs. Superior phase-change properties of AMS based on amorphous and crystalline phase-change process is similar to the origins of high device performance in the case of the [GeTe/Sb_2_Te_3_]_n_ superlattice. However, AMS is easier to fabricate and initialize after amorphization, compared to the sophisticated growth conditions of the [GeTe/Sb_2_Te_3_]_n_ superlattice. Moreover, density of AC and QC is 3.95 and 4.03, respectively, where even a miniscule change in volume (2%) during phase-change process helps to secure cyclability of the cell, indicating that AMS is optimized to be commercialized as PCMs in 3 dimensional device technology. Development of new PCMs based on phase-change processes from QC to AC is expected with increased resistivity and enhanced contrast in electrical resistance. The QCs proposed in this study represent a promising alternative to GST for next-generation PCMs. The development of new PCMs based on the phase change process from QC to AC is expected to be possible with QC materials that are well-designed for PCM.

## Methods

### Sample preparation and characterization

Alloys containing 10–25 at.% Si, 10—25 at.% Mn, and remainder Al were prepared by melting mixtures of high purity elements under an Ar atmosphere using high-frequency induction. Ribbon-type samples of ~ 20 μm thickness were prepared for phase identification (Fig. 1) using the melt spinning method on a copper wheel (of diameter 22 cm) rotating at 3,000 rpm. The crystal structure of the as-melt-spun samples was analyzed using an x-ray diffractometer (XRD, D8 Advance, BRUKER) with Cu K_α_ radiation. Thin foil TEM samples were prepared using a focused ion beam (FIB) microscope (Helios NanoLab 600, FEI) and then mounted onto a micro-electromechanical systems-based (MEMS-based) chip for in-situ heating during TEM (Fusion Select, Protochips). The local structure was investigated using a probe aberration-corrected scanning TEM (STEM) system (Titan™ 80–300, FEI) at an acceleration voltage of 300 keV. The evolution of the elemental composition was tracked using STEM (TalosF200X, FEI) equipped with an X-FEG source and Super-X EDS (Bruker) with a quadruple detector. The thermal behavior of the as-melt-spun samples was analyzed using differential scanning calorimeters (DSCs, NETZSCH STA 449 F5 JUPITER for high temperatures up to 1,100 K and Perkin Elmer DSC 8,000 for low temperatures up to 900 K). Thin Al–Mn–Si films were deposited by direct current and radio frequency co-sputtering of high purity Al, Si, and Mn (99.999%) on SiO_2_/Si (100) wafer substrates at room temperature. The pressure inside the chamber was less than 2.0 × 10^−7^ T. All of the deposition processes were carried out in an Ar atmosphere at 3 × 10^−3^ T.

### Resistance–temperature measurements and characterization of cell performance

The dependence of temperature on resistivity was measured using the two point probe method with prepared samples. These samples consisted of Al_60_Mn_20_Si_20_ films of 50, 100, and 200 nm thickness that were deposited on a thermally oxidized 100 nm SiO_2_ on Si wafer and encapsulated with a 5 nm layer of SiO_2_ to avoid oxidation during the annealing process. The as-grown films were in the amorphous phase regardless of their Al–Mn–Si composition due to their extremely fast cooling speed (~ 10^12^ K/s) during sputtering deposition, as depicted in Supplement Fig. S2. In order to form the QC phase, the thin films were annealed at 623 K for 2 h in an Ar atmosphere prior to the measurement of the dependence of resistance on temperature during the annealing and cooling processes. Additionally, to compare the fabricated material with\conventional PCMs, 100 nm thick Ge_2_Sb_2_Te_5_ films were also deposited on SiO_2_ wafers and also encapsulated with a 5 nm layer of SiO_2_ using the ion beam sputtering deposition method at room temperature under a 3 × 10^−8^ T vacuum. The resistivity was measured by gradually increasing the temperature from room temperature to 813 K during the annealing process and subsequently decreasing the temperature to room temperature during cooling process, where the annealing and cooling rates were both 30 K/min. Ar purging was performed five times after evacuation until 5 × 10^−4^ T vacuum was achieved before measurement and an Ar atmosphere was maintained throughout the measurement process.

T-shaped Al–Mn–Si alloy and GST test cells were fabricated to investigate the electrical phase change characteristics. The BEC was patterned using photolithography, was composed of TiN, and was 300 nm in diameter. Co-sputtering Al, Si, and Mn targets were used in the RF sputtering method to form a 100 nm thick Al–Mn–Si layer and a single GST target was used in the ion-beam sputtering method to form a GST layer of the same thickness. Above the PCM films, a 50 nm thick TiN layer was deposited using the RF-sputtering method to form the top electrode. An Agilent 33600A pulse generator and a Keithley 2636B source meter were used to apply the electrical pulses and to measure the changes in resistance. To change the resistance levels for the set and reset operations, electrical pulses of 10–200 ns width and of 2–6 V magnitude were applied. The resistance of each AC and QC phase was measured using a 0.1 V probing voltage.

### Optical carrier dynamics measurements

For the THz-TDS measurements, Al–Mn–Si samples of 50 nm thickness were sputtered onto a sapphire substrate using the same condition as that for thin-film deposition. To obtain the QC and AC samples, the samples were annealed at 350 °C for 2 h and at 600 °C for 30 min, respectively. Terahertz spectroscopy probes were used to measure the electromagnetic response of free carrier and phonons in materials in the energy range from 0.8 to 8.7 meV, where we study carrier density, effective mass, and the scattering rate of free carriers.

The THz transmission spectra were obtained using a standard THz-TDS setup^37^. A femtosecond Ti:sapphire laser (Mai Tai, Spectra-physics) was used with a pulse of 35 fs width and 1 kHz repetition rate. The laser was divided into generation and detection parts; a ZnTe (110) crystal of 3 mm thickness was used for optical rectification generation, and a ZnTe crystal of 2 mm thickness was used for electro-optical sampling detection. The THz probe beam was 1 mm in diameter (full width at half maximum, FWHM). Normalization against the substrate response was achieved using a separate measurement recorded using a bare substrate. Internal reflections in substrate were excluded and time-domain signals were then Fourier-transformed to obtain energy (frequency)-dependent spectral functions. Complex transmittance was normalized and converted to complex conductance spectra based on Tinkham’s formula^38^, which is valid for the ultrathin-film limit studied here. This formula is given by t_fs_/t_s_ = 1/[1 + Z_0_G/(η_s_ + 1)], where *t*_*fs*_ and *t*_*s*_ are the complex transmission coefficients of the sample (film plus substrate) and the bare substrate, respectively. In addition, *Z*_*0*_ is the vacuum impedance, *G* is the conductance of the film, and *η*_*s*_ is the refractive index of the bare substrate. For bulk free-carrier states, *G* = *σd,* where *σ* is the optical conductivity and *d* is the thickness of the film.

## Supplementary information

Supplementary Information.

Supplementary Video 1.

## Data Availability

The data sets generated and/or analyzed during this study are available from the corresponding author on reasonable request.

## References

[1] Ovshinsky SR (1968). Reversible electrical switching phenomena in disordered structures. Phys. Rev. Lett..

[2] Yamada N (1987). High speed overwritable phase change optical disk material. Jpn. J. Appl. Phys..

[3] Iwasaki H, Ide Y, Harigaya M, Kageyama Y, Fujimura I (1992). Completely erasable phase change optical disk. Jpn. J. Appl. Phys..

[4] Zhu M (2014). One order of magnitude faster phase change at reduced power in Ti–Sb–Te. Nat. Commun..

[5] Zhou Y (2017). Reducing the stochasticity of crystal nucleation to enable subnanosecond memory writing. Science (80-).

[6] Kolobov AV (2004). Understanding the phase-change mechanism of rewritable optical media. Nat. Mater..

[7] Wuttig M (2007). The role of vacancies and local distortions in the design of new phase-change materials. Nat. Mater..

[8] Matsunaga T (2011). From local structure to nanosecond recrystallization dynamics in AgInSbTe phase-change materials. Nat. Mater..

[9] Zhu M (2019). Direct atomic insight into the role of dopants in phase-change materials. Nat. Commun..

[10] Rao F (2015). Direct observation of titanium-centered octahedra in titanium–antimony–tellurium phase-change material. Nat. Commun..

[11] Baeck JH (2009). Electronic structure of Te/Sb/Ge and Sb/Te/Ge multi layer films using photoelectron spectroscopy. J. Am. Chem. Soc..

[12] Park SJ (2015). Laser irradiation-induced modification of the amorphous phase in GeTe films: The role of intermediate Ge–Te bonding in the crystallization mechanism. J. Mater. Chem. C.

[13] Jang MH (2010). The origin of the resistance change in GeSbTe films. Appl. Phys. Lett..

[14] Xia Q, Yang JJ (2019). Memristive crossbar arrays for brain-inspired computing. Nat. Mater..

[15] Simpson RE (2011). Interfacial phase-change memory. Nat. Nanotechnol..

[16] Shechtman D, Blech I, Gratias D, Cahn JW (1984). Metallic phase with long-range orientational order and no translational symmetry. Phys. Rev. Lett..

[17] Tsai AP (2008). Icosahedral clusters, icosaheral order and stability of quasicrystals-A view of metallurgy. Sci. Technol. Adv. Mater..

[18] Widom M, Strandburg KJ, Swendsen RH (1987). Quasicrystal equilibrium state. Phys. Rev. Lett..

[19] Kamiya K (2018). Discovery of superconductivity in quasicrystal. Nat. Commun..

[20] Tang Y (2019). Quasicrystal photonic metasurfaces for radiation controlling of second harmonic generation. Adv. Mater..

[21] Kirihara K, Kimura K (2002). Composition dependence of thermoelectric properties of AlPdRe icosahedral quasicrystals. J. Appl. Phys..

[22] Elser V (1985). Indexing problems in quasicrystal diffraction. Phys. Rev. B.

[23] Chen CH, Chen HS (1986). ‘Superlattices’ in quenched Al–Si–Mn quasicrystals. Phys. Rev. B.

[24] Socolar JES, Lubensky TC, Steinhardt PJ (1986). Phonons, phasons, and dislocations in quasicrystals. Phys. Rev. B.

[25] Fung KK, Zhou YQ (1986). Direct observation of the transformation of the icosahedral phase in (Al_6_mn)_1–x_Si_x_, into α(almnsi). Philos. Mag. B Phys. Condens. Matter. Stat. Mech. Electron. Opt. Magn. Prop..

[26] Orava J, Greer AL, Gholipour B, Hewak DW, Smith CE (2012). Characterization of supercooled liquid Ge2Sb2Te5and its crystallization by ultrafast-heating calorimetry. Nat. Mater..

[27] Tsai AP, Hiraga K, Inoue A, Masumoto T, Chen HS (1994). Annealing-induced icosahedral glass phase in melt-spun Al–Cu–V and Al–Si–Mn alloys. Phys. Rev. B.

[28] Turnbull D (1950). Formation of crystal nuclei in liquid metals. J. Appl. Phys..

[29] Lee B (2009). Observation of the role of subcritical nuclei in crystallization of a glassy solid. Science (80-).

[30] Lee BS (2014). Nanoscale nuclei in phase change materials: Origin of different crystallization mechanisms of Ge2Sb2Te5 and AgInSbTe. J. Appl. Phys..

[31] Kimura K, Hahimoto T, Sujuki K, Nagayama K, Ino H, Takeuchi S (1986). Structure and stability of quasicrystalline Al–Mn alloys. J. Phys. Soc. Jpn..

[32] Kalb JA, Wuttig M, Spaepen F (2007). Calorimetric measurements of structural relaxation and glass transition temperatures in sputtered films of amorphous Te alloys used for phase change recording. J. Mater. Res..

[33] Takagiwa Y, Kimura K (2014). Metallic-covalent bonding conversion and thermoelectric properties of Al-based icosahedral quasicrystals and approximants. Sci. Technol. Adv. Mater..

[34] Kirihara K (2000). Covalent bonds in AlMnSi Icosahedral quasicrystalline approximant. Phys. Rev. Lett..

[35] Kirihara K, Kimura K (2000). Covalency, semiconductor-like and thermoelectric properties of Al-based quasicrystals: Icosahedral cluster solids. Sci. Technol. Adv. Mater..

[36] Steurer W (2005). Structural phase transitions from and to the quasicrystalline state. Acta Crystallogr. Sect. A Found. Crystallogr..

[37] Maeng I (2019). Significant THz absorption in CH_3_ NH_2_ molecular defect-incorporated organic-inorganic hybrid perovskite thin film. Sci. Rep..

[38] Choi H (2015). Evolution of the surface state in Bi_2_Se_2_Te thin films during phase transition. Nanoscale.

