# Supplementary material for: Quasicrystalline phase-change memory
Source: Sci Rep. 2020 Aug 13;10:13673. doi: 10.1038/s41598-020-70662-2 (PMC7426956; doi:10.1038/s41598-020-70662-2)
Supplement: Supplementary file 1 — Supplementary Information. [file 41598_2020_70662_MOESM1_ESM.docx]

Supporting Information

**Quasicrystalline Phase-Change Memory**

Eun-Sung Lee^[[1]](#footnote-1),,†, *^, Joung E. Yoo^1,†^, Du S. Yoon^1,†^, Sung D. Kim^1^, Yongjoo Kim^2^, Soobin Hwang^3^, Dasol Kim^3^, Hyeong-Chai Jeong^4^, Won T. Kim^5^, Hye J. Chang^6^, Hoyoung Suh^6^, Dae-Hong Ko^2^, Choonghee Cho^2^, Yongjoon Choi^2^, Do H. Kim^2,*^, and Mann-Ho Cho^3,*^

*^1^* Material Research Center, SAIT, Samsung Electronics, Suwon, 16678, Republic of Korea^2^ Department of Materials Science and Engineering, Yonsei University, Seoul, 03722, Republic of Korea ^3^ Department of Physics, Yonsei University, Seoul, 03725, Republic of Korea ^4^ Department of Physics and Astronomy, Sejong University, Seoul, 05006, Republic of Korea ^5^ Department of Optical Engineering, Cheongju University, Cheongju, 28503, Republic of Korea ^6^ Advanced Analysis Center, Korea Institute of Science and Technology, Seoul, 02792, Republic of Korea. *^†^*These authors contributed equally to this work. *^*^*Correspondence and requests for materials should be addressed to M.H.C. (email: [mh.cho@yonsei.ac.kr](mailto:mh.cho@yonsei.ac.kr)), D.H.K. (email: [dohkim@yonsei.ac.kr](mailto:dohkim@yonsei.ac.kr)), or E.S.L. (email: [e.lee@samsung.com](mailto:e.lee@samsung.com)).

The supplementary information provides additional information to accompany that in the article. Transmission electron microscopy (TEM), differential scanning calorimetry (DSC), X-ray diffraction (XRD), Terahertz time domain spectroscopy (THz-TDS) are included. Supplementary Video SV1 and Supplementary Figure S4 provide results obtained by TEM for structural analysis. Supplementary Figure S2 provides specific results obtained by DSC for activation energy corresponding to transition from quasi-crystalline (QC) to approximant crystalline (AC) phase. Supplementary Figure S3 provides results obtained by XRD to identify structural difference between amorphous, QC, and AC phase. Supplementary Figure S5 provides results obtained by THz-TDS to study electrical properties.

**Supplementary Video SV1.** Video of the in-situ TEM observation shown in Fig 2. (d)–(g). Repository will be provided.

**
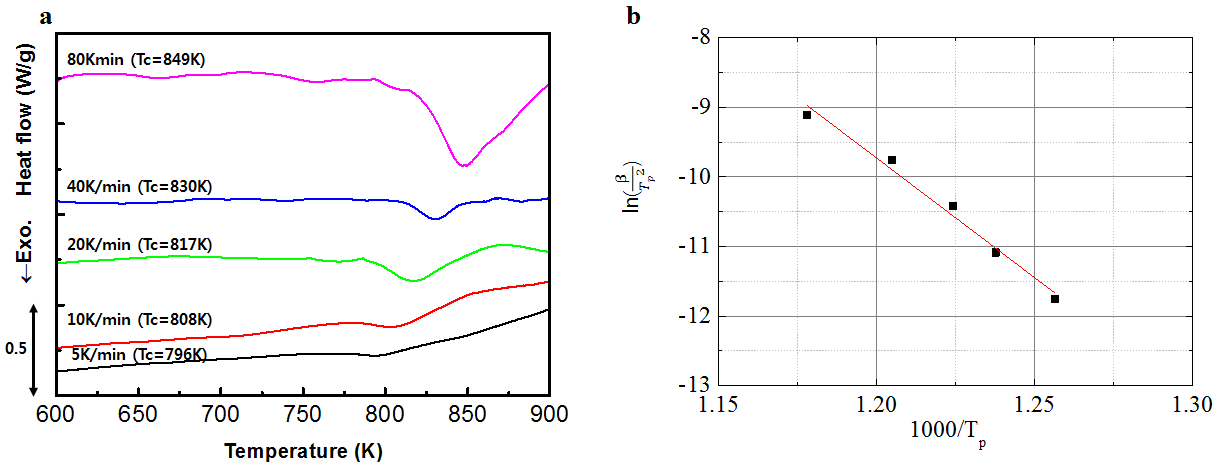
**

**Supplementary Figure S2.** DSC and activation energy obtained by fitting model with Arrhenius plot. Activation energy is 2.96eV within the margin of error of 0.21eV.

**
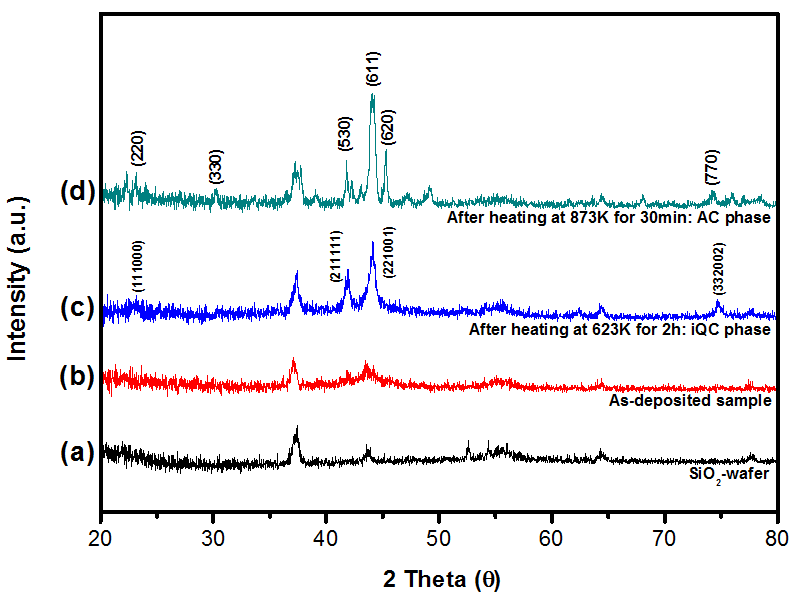
**

**Supplementary Figure S3.** X-ray diffraction data for: (a) SiO_2_ wafer; (b) Al_60_Mn_20_Si_20_ as-deposited sample (200 nm thick on SiO_2_ wafer); (c) after heating to 623 K for 2h; and (d) after heating to 873 K for 30min. The as-deposited sample consisted of the amorphous phase. After heating to 623 K, the amorphous phase transformed into the QC phase. After heating to 873 K for 30min, the QC phase transformed into the AC phase.

**
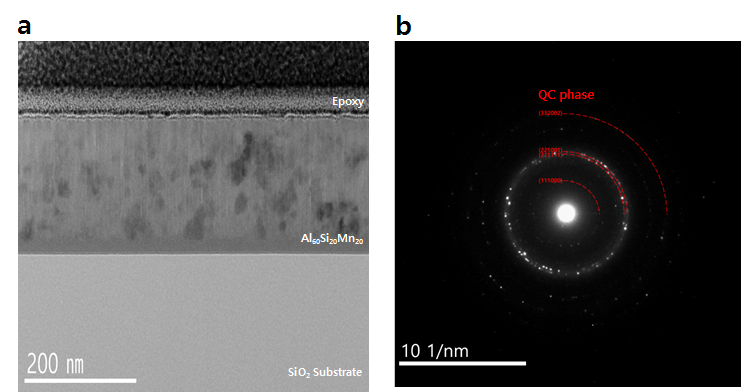
**

**Supplementary Figure S4.** (a) BF image and (b) SADP obtained for the 200 nm thick Al_60_Mn_20_Si_20_ sample on a SiO_2_ wafer after heating at 623 K for 2 h.

**
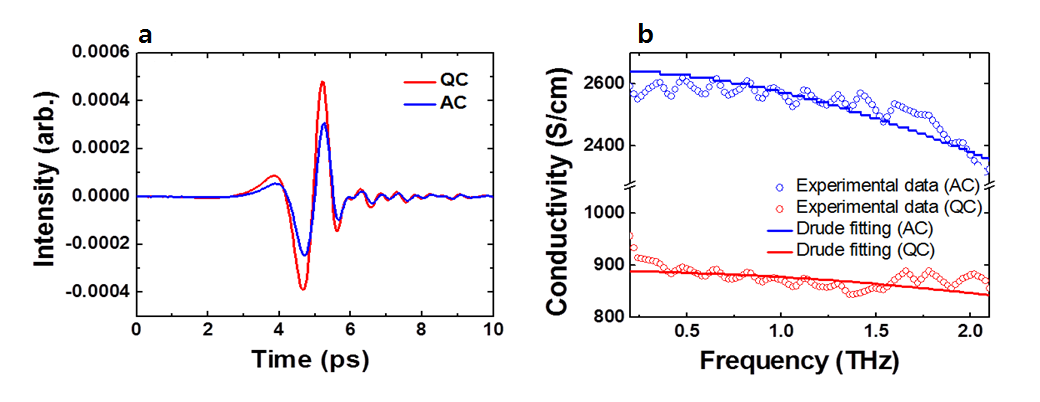
**

**Supplementary Figure S5.** Terahertz pulses and real conductivity of QC and AC. (a) THz-TDS pulse for QC (red line) and AC (blue line). The carrier density is larger in the AC than the QC. (b) Frequency-dependent real conductivity of QC and AC converted from the TDS spectrum of each sample. Drude model fitting is also shown. The electron plasma frequencies (*ω_p_*) were 0.5 eV (QC) and 0.7 eV (AC). The electron scattering rates (*γ_0_*) were 9×10^12^ s^−1^ (QC) and 6×10^12^ s^−1^ (AC).

1. *^1^* Material Research Center, SAIT, Samsung Electronics, Suwon, 16678, Republic of Korea^2^ Department of Materials Science and Engineering, Yonsei University, Seoul, 03722, Republic of Korea ^3^ Department of Physics, Yonsei University, Seoul, 03725, Republic of Korea ^4^ Department of Physics and Astronomy, Sejong University, Seoul, 05006, Republic of Korea ^5^ Department of Optical Engineering, Cheongju University, Cheongju, 28503, Republic of Korea ^6^ Advanced Analysis Center, Korea Institute of Science and Technology, Seoul, 02792, Republic of Korea. *^†^*These authors contributed equally to this work. *^*^*Correspondence and requests for materials should be addressed to M.H.C. (email: [mh.cho@yonsei.ac.kr](mailto:mh.cho@yonsei.ac.kr)), D.H.K. (email: [dohkim@yonsei.ac.kr](mailto:dohkim@yonsei.ac.kr)), or E.S.L. (email: [e.lee@samsung.com](mailto:e.lee@samsung.com)). [↑](#footnote-ref-1)
